# Absent right superior vena cava

**DOI:** 10.1002/jgf2.419

**Published:** 2021-01-25

**Authors:** Takahiro Hayashi, Masato Murakami, Shigeru Saito

**Affiliations:** ^1^ Department of Cardiology Shonan Kamakura General Hospital Kamakura Japan

**Keywords:** congenital heart disease, isolated persistent left superior vena cava

## Abstract

A patient planned to be performed catheter ablation. However, three‐dimensional contrast‐enhanced chest computed tomography revealed isolated persistent left superior vena cava. We should know such an anatomical abnormality especially when central venous catheter or peripherally inserted central catheter is inserted from right jugular vein or right subclavian vein

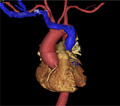

A 58‐year‐old man was scheduled to undergo catheter ablation for paroxysmal supraventricular tachycardia. However, an anatomical abnormality of the superior vena cava was suspected when the electrode catheter was inserted from the right jugular vein. Simultaneous angiography of the right subclavian vein and right atrium showed the absence of the superior vena cava on the right side (Video S1). Three‐dimensional contrast‐enhanced chest computed tomography revealed that a persistent left superior vena cava (Figure 1, blue vessel) led to a dilated coronary sinus (Figure 2, blue vessel). The absence of the superior vena cava on the right side and its persistence on the left confirmed the diagnosis of an isolated persistent left superior vena cava.

**FIGURE 1:**
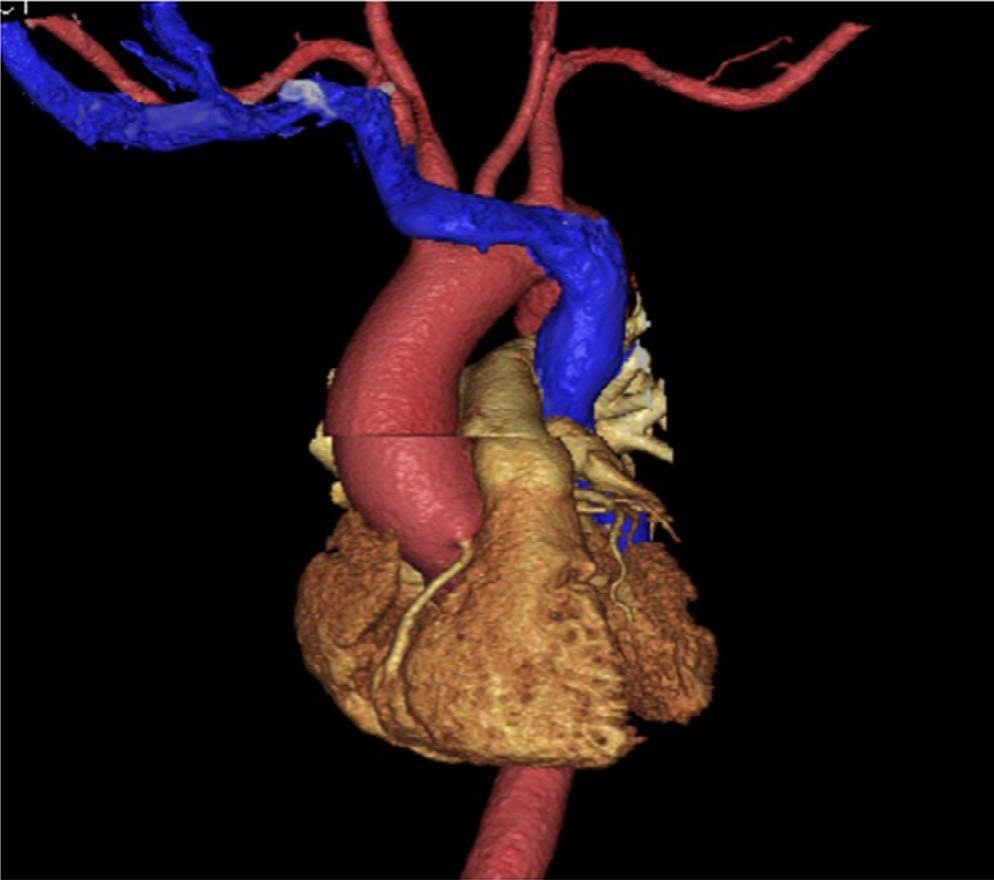
A three‐dimensional CT image (anterior view): A persistent left superior vena cava without a superior vena cava on the right. CT, computed tomography

**FIGURE 2:**
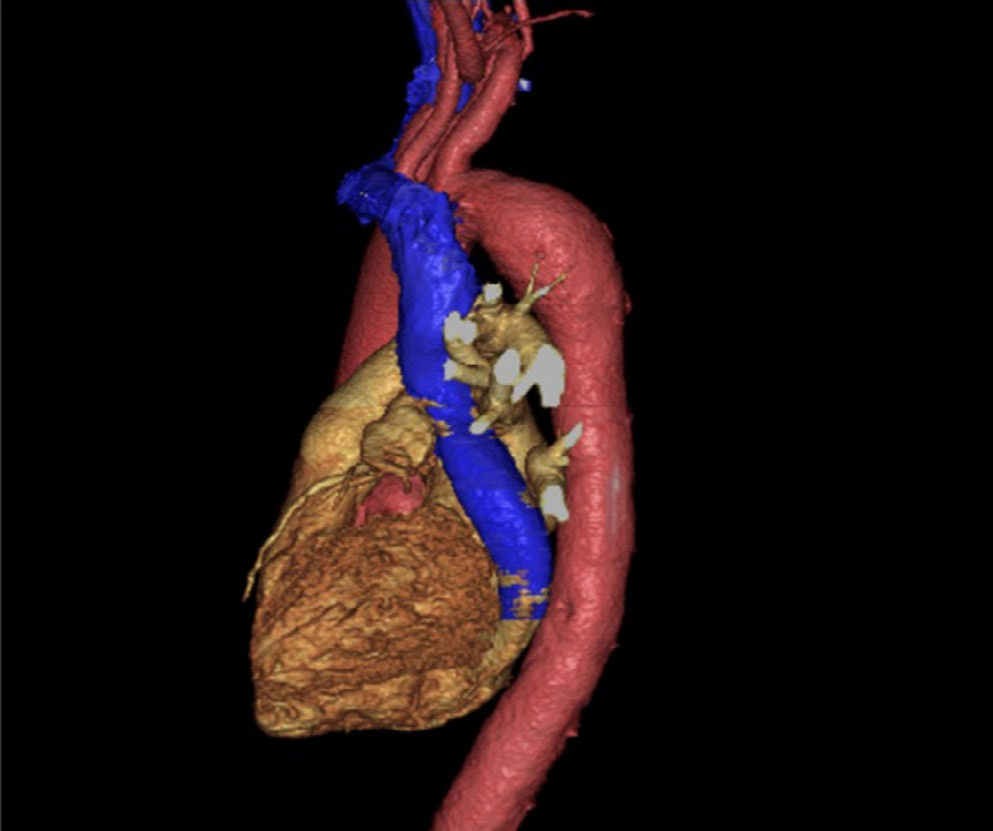
A three‐dimensional CT image (lateral view): A persistent left superior vena cava leading to the right atrium via a dilated coronary sinus. CT, computed tomography

Isolated persistent left superior vena cava occurs in only 0.09%–0.13% of the patients with congenital heart disease.^1^ We should keep such an anatomical abnormality in mind, especially when a central or a peripherally inserted central venous catheter is inserted into the right jugular vein or the right subclavian vein.

## CONFLICT OF INTEREST

The other authors have stated explicitly that there are no conflicts of interest in connection with this article.

## INFORMED CONSENT

We have obtained the consent of the patient for publication.

## Supporting information

Video S1
